# Patient-specific drill template for C2 transoral pedicle insertion in complete reduction of atlantoaxial dislocation: cadaveric efficacy and accuracy assessments

**DOI:** 10.1186/s13018-019-1189-7

**Published:** 2019-05-16

**Authors:** Lijun Lin, Meisong Zhu, Peng Peng, Xintao Zhang, Xiaoqi Zhou, Jianyi Li

**Affiliations:** 1 Department of Orthopedics, Zhujiang Hospital, Southern Medical University, 253 Gongye Street, Haizhu District, Guangzhou, Guangdong China; 2 Department of Anatomy, Guangdong Provincial Key Laboratory of Medical Biomechanics, School of Basic Medical Sciences, Southern Medical University, 1063 Shatai Nan Road, Baiyun District, Guangzhou, Guangdong China; 3 Department of Radiology, The Third Affiliated Hospital, Southern Medical University, 183 Zhongshandadao Xi, Tianhe District, Guangzhou, Guangdong China; 4 Nanhai Hospital, Southern Medical University, 28 Liguan Road, Lishui Town, Nanhai District, Foshan, Guangdong China

**Keywords:** Patient-specific drill template, Transoral atlantoaxial reduction plate, Atlantoaxial dislocation, Pedicle fixation

## Abstract

**Background:**

The transoral atlantoaxial reduction plate (TARP) is an effective advance in the treatment of atlantoaxial dislocation (AAD) and can enable the performance of anterior atlantoaxial release, reduction, decompression, and internal fixation in a one-stage operation. However, accurate transoral C2 pedicle insertion (C2TOPI) remains a challenge. The aim of this study is to develop a grouped patient-specific drill template (PDT) specifically for AAD with complete reduction and, furthermore, to compare its efficacy and accuracy in facilitating C2TOPI.

**Methods:**

After CT scanning, ten cadaveric C2 specimens were randomly assigned to two groups (the PDT and freehand group). A grouped PDT specifically for AAD with complete reduction was designed and manufactured. C2TOPI was performed using the PDT or the fluoroscopy-guided freehand technique. Postoperative CT scans were subsequently performed to analyze the deviations at the centroid of the cross section at the midpoint of the pedicle. Screw position grades were also assessed in both groups.

**Results:**

Compared to the freehand group, the PDT group had a significantly shorter surgery time (*p* < 0.001). Significant differences between the two groups were observed in the absolute value of the deviations at the centroid of the pedicle on either the axial or sagittal planes (*p* < 0.05). No significant difference was found in the screw positions between the two groups (*p* > 0.05); however, two unacceptable breaches (20%) occurred in the freehand group.

**Conclusion:**

A specifically designed PDT could provide an accurate and easy-to-apply method for C2TOPI in AAD with complete reduction.

## Background

The surgical treatment of atlantoaxial dislocation (AAD) presents a difficult surgical challenge for neurospinal surgeons [1, 2]. A transoral atlantoaxial reduction plate (TARP) was developed specially for AAD and became an effective advance in the treatment of AAD [3–5] that could allow anterior atlantoaxial release, reduction, decompression, and internal fixation in a one-stage operation. Transoral C2 pedicle fixation was adopted in the third generation of the TARP system [6], which could effectively improve screw anti-pullout force and might offer higher stability than intravertebral insertion fixation. However, due to anatomical variation and the proximity to the vertebral artery and spinal cord, transoral C2 pedicle insertion (C2TOPI) is relatively difficult and has a high potential for injury of the arteries, nerve roots, and the dural sac [7].

Accurate C2TOPI is a key to successful clinical application of the TARP system. Because of the anatomical complexity of atlantoaxial structures and the area covered by the TARP, C2TOPI is a relatively difficult task. The anatomical freehand technique remains the mainstay in clinical practice. However, screw malposition occurs frequently [8–10]. C-arm fluoroscopy was thus introduced to facilitate pedicle screw placement in upper cervical spinal surgeries [11, 12]. However, the overlapping images are not sufficient for atlantoaxial complexity and therefore cannot indicate the screw position precisely [13]. Li et al. [2] reported the placement of C2TOPI using the freehand technique under C-arm fluoroscopy, in which 46.9% of screw threads penetrated the bony cortex of pedicles. Further research revealed that the pedicle cortex penetration rate in unacceptable positions was as high as 13.0%; one patient died postoperatively as a result of C2 screw misplacement [1]. Furthermore, intraoperative fluoroscopy might extend the operation time and increase the total amount of radiation; this is dangerous for both the patient and the surgical team [14, 15].

Patient-specific drill templates (PDTs) produced by three-dimensional (3D) printing have been developed for spinal surgeries; this method could not only obviate radiation exposure and time-consuming procedures, but could also improve the accuracy in cervical posterior pedicle insertions [16–20]. However, PDTs for C2TOPI are rare. To the best of our knowledge, only Li et al. [2] reported grouped PDTs in their cadaveric study, in which several graded screw trajectories were pre-set to facilitate C2TOPI. However, because the intraoperative reduction of AAD might not be perfectly fit for the pre-set graded screw trajectories, potential risks could be present in the clinical application of PDTs.

Intraoperative reduction of AAD can be divided into two situations, complete and incomplete reductions [4, 21], which require different strategies for PDT design. In this study, we developed a grouped PDT specifically for AAD with complete reduction and compared its efficacy and accuracy in facilitating C2TOPI. We hypothesized that this grouped PDT might be an alternative to the fluoroscopy-guided freehand technique for C2TOPI.

## Materials and methods

### Specimens

Ten formalin-preserved human cadaveric cervical spines (six males and four females, from 48 to 68 years of age, mean 62.5 years) were obtained. The transverse ligament and other soft tissues around the odontoid were resected to create simulated AADs between C1 and C2. The study used a randomized double-blind design. After the anterior soft tissue of C1 and C2 was removed from the vertebrae, 20 sides of ten formalin-preserved cadaveric cervical spines were randomly divided into two groups, the PDT and freehand group, using a random number chart. The internal atlantoaxial fixations in all specimens were performed using the fourth generation of the TARP system that added the fixation using vertebral body screws (VBSs) based on the third generation of the TARP system [21].

### Optimal trajectories of C2TOPI for AAD with complete reduction

**Fig. 1:**
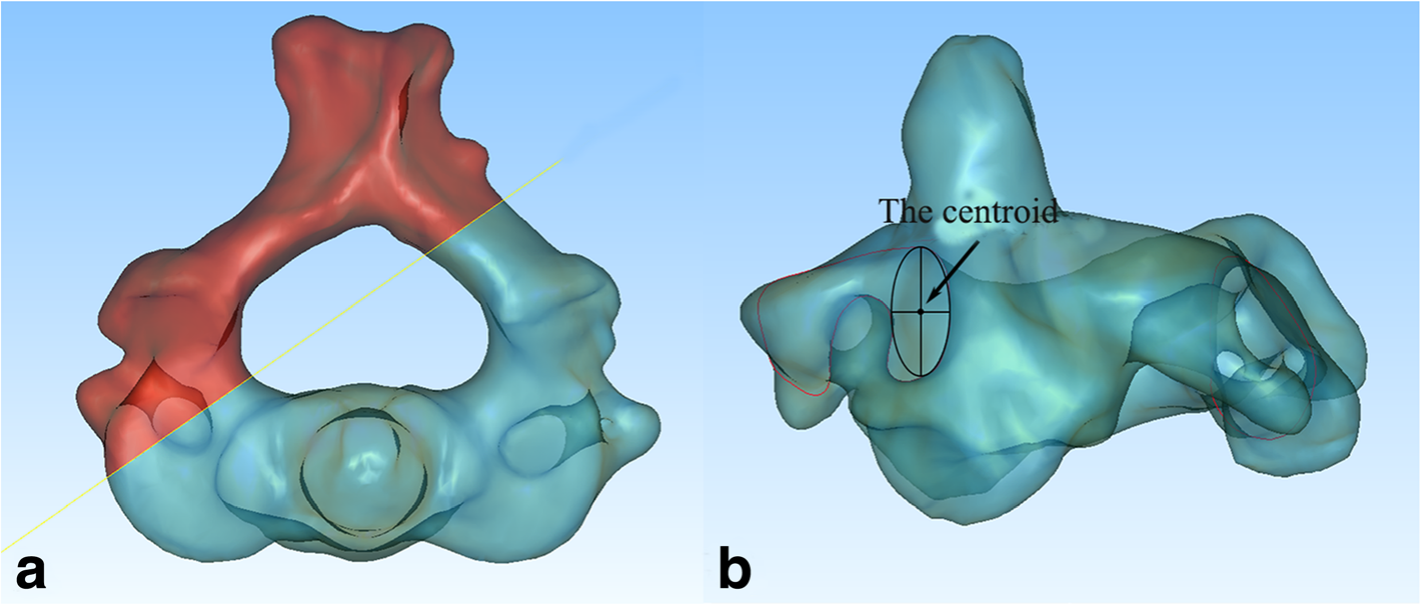
Calculation of the centroid of the cross section at the midpoint of the pedicle of C2. **a** The midpoint and corresponding cross section of each pedicle were determined. **b** The centroid was calculated with the contour of the cross section at the midpoint of the pedicle

All specimens were scanned by a Brilliance CT 64-channel scanner (Philips, Eindhoven, The Netherlands) in 0.625 mm intervals with a pixel size of 0.55 mm. All cervical vertebrae involved in this study showed no bone defects or fractures according to the CT scan images. The CT data were imported into Mimics software version 14.11 (Materialise Corp., Leuven, Belgium) to obtain 3D reconstruction models of the C1 and C2 vertebrae. The 3D model of C2 was then exported to Geomagic Studio software, version 11.0 (3D Systems Corp, Morrisville, NC), to calculate the centroid of the cross section at the midpoint of the pedicle (Fig. 1).

**Fig. 2:**
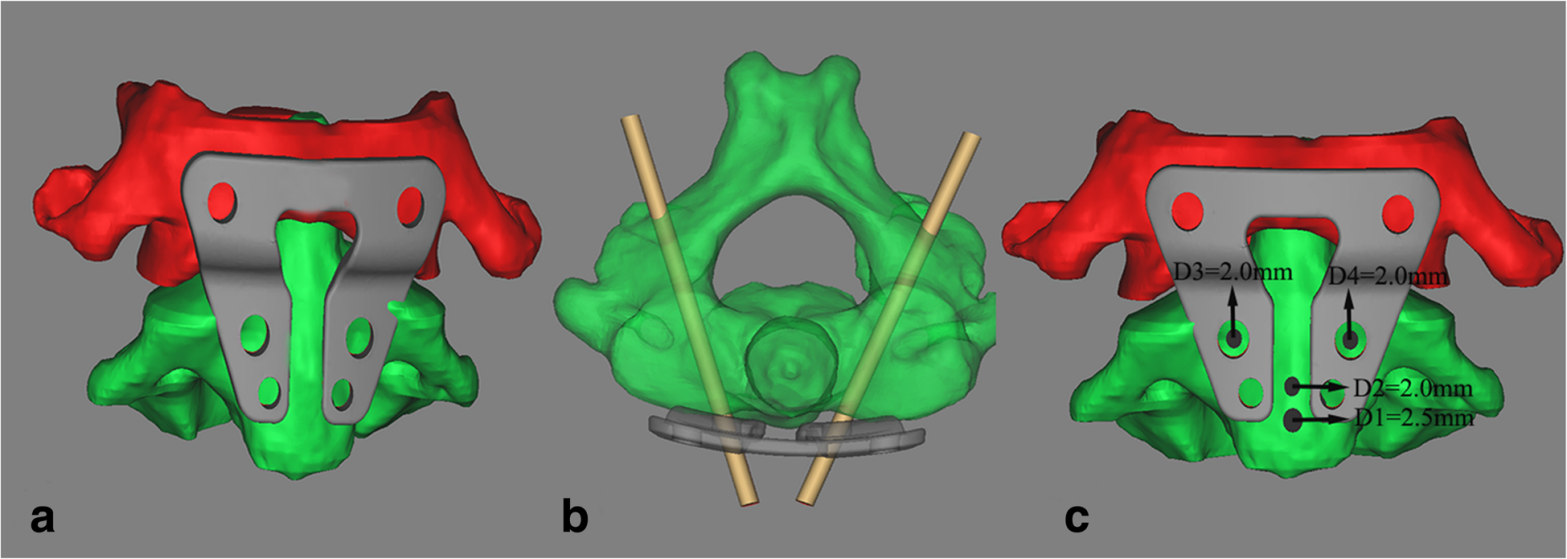
Preoperative plan for TARP position and optimal trajectories. **a** After virtual reduction of C1 and C2, the TARP was moved to a proper position to simulate C1/2 fixation, in which the entrance point of the vertebral body was determined. **b** The optimal trajectory was determined by the entrance point of the vertebral body and the centroid at the midpoint of the pedicle. **c** Four additional holes were set. D1 was a 2.5-mm-diameter hole for a temporary reduction screw (TRS) that was as high as the inferior border of the TARP. D2 was a 2.0-mm-diameter hole for a setscrew that was 5 mm higher. D3 and D4 were 2.0-mm-diameter holes for the entry points of the trajectory for C2TOPI, which were located at the center of the pedicle screw holes of the TARP

3D models of C1, C2, and the TARP were imported into Solidworks 2014 (Dassault Systemes, France). After virtual reduction of C1 and C2, the TARP was moved to a proper position (complete anatomic reduction) to simulate C1/2 fixation, in which the entry points of the vertebral body were determined (Fig. 2a). The optimal trajectory was determined by the entry point and the centroid at the midpoint of the pedicle, in which the medial wall of the transverse foramen and the lateral wall of the spinal canal were also considered (Fig. 2b). Subsequently, four additional holes were set. D1 was a 2.5-mm-diameter hole for a temporary reduction screw (TRS) that was as high as the inferior border of the TARP. D2 was a 2.0-mm-diameter hole for a setscrew that was 5 mm higher. D3 and D4 were 2.0-mm-diameter holes for trajectory entry points for C2TOPI that were located at the center of the pedicle screw holes of the TARP (Fig. 2c). Furthermore, D1 and D2 were used for the placement of PDT B in the procedure, and D1, D3, and D4 assisted the TARP to find the ideal trajectory position of C2TOPI.

### Design and manufacturing of PDTs for C2TOPI

**Fig. 3:**
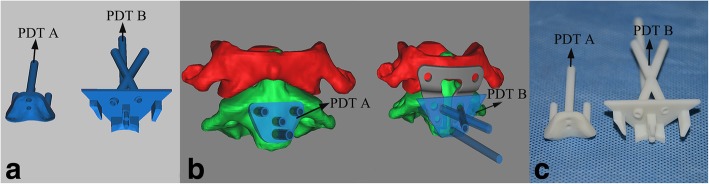
Grouped PDT of C2TOPI for AAD with complete reduction. **a** Computer-aided-designed models of PDT A and B. **b** Simulations of C2TOPI with PDT A and B. **c** 3D-printed models of PDT A and B

Following the pre-set four holes and C2TOPI trajectory, a grouped PDT for C2TOPI was specially designed and had two parts, referred to as PDT A and B (Fig. 3). PDT A was used to guide the four preoperatively designed holes (D1, D2, D3, and D4) and had a bottom surface and four guide tubes. The bottom surface was the reverse surface of the anterior surface of the vertebral body, by which PDT A could tightly attach to C2. PDT B was used for C2TOPI drilling. Since the TARP covered most areas of the anterior surface of C2 in the operative procedure, a special “table” structure was designed. The bottom surfaces of the columns under the table were aligned to the surface of C2 that was not covered by the TARP. The two short pins extended from the middle columns which were aligned to the two holes for the TRS and setscrew (D1 and D2). The table included two drilling tubes based on the planned trajectories. PDT A and B were both 3D printed by a RS6000 stereolithography printer (Shanghai Union Technology Corp, Shanghai, China) (Fig. 3).

### Cadaveric surgery for C2TOPI

**Fig. 4:**
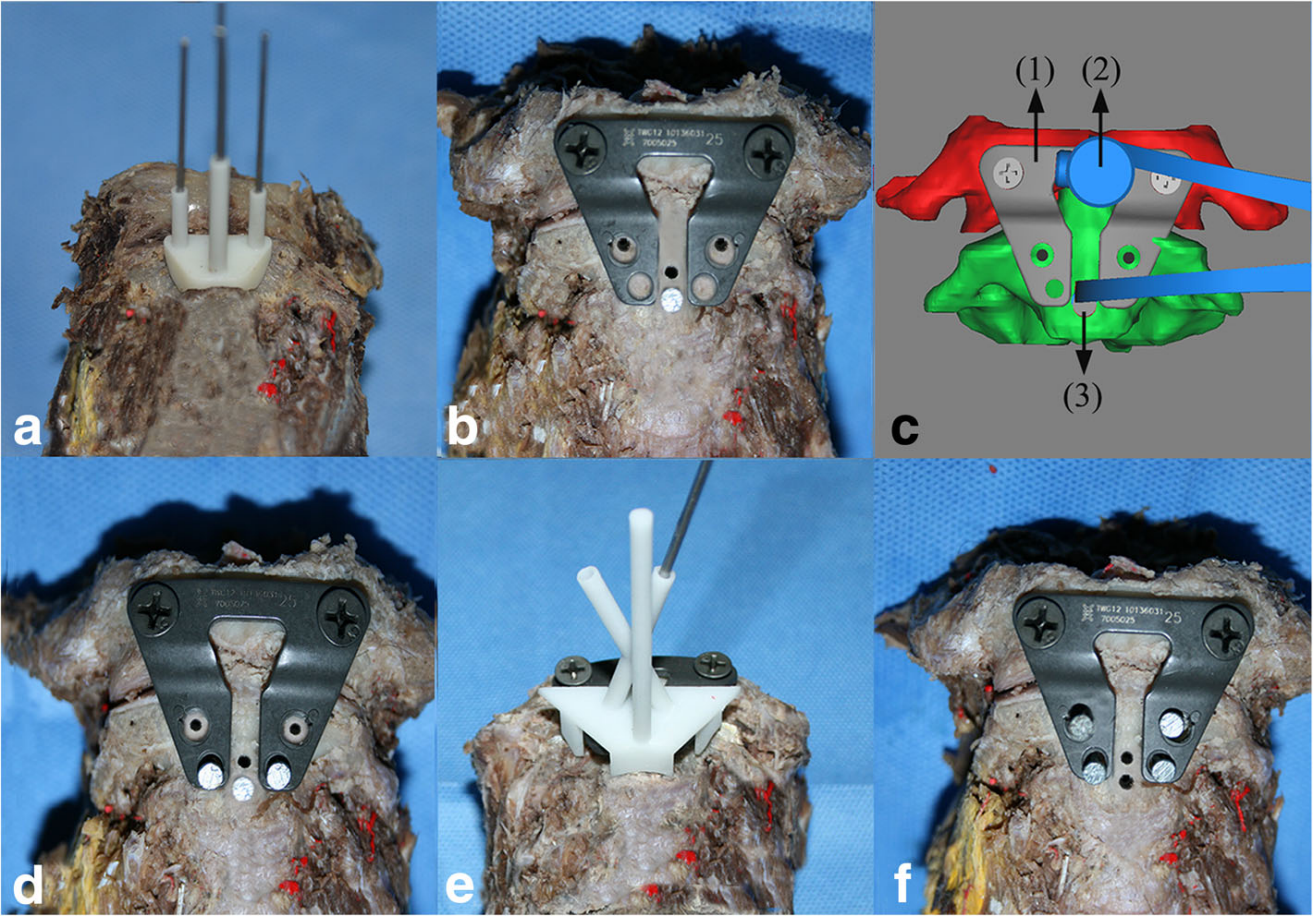
Cadaveric C2TOPI with PDT A and B. **a** Three 2.0-mm-diameter and one 2.5-mm-diameter K-wires were inserted through the guide tubes of the PDT A. **b** A TRS was then inserted. The upper two holes of the TARP were fixed to C1 with lateral mass screws. **c** A special reduction instrument was used to relocate C1 and C2 by applying force between the TARP and TRS. In the figure, (1) indicates the TARP, (2) indicates the reduction instrument, and (3) indicates the TRS. **d** When the inferior border of the TARP reached the midpoint of the TRS and the two entry points of C2TOPI were detected at the center of the pedicle screw holes of the TARP, the inferior two holes of the TARP were fixed to C2 with VBSs. **e** After removing the TRS, a K-wire was inserted into the pedicle with the assistance of the PDT B. **f** Two 3.5-mm-diameter screws were inserted

An attending spinal surgeon was asked to perform the C2TOPI with either the PDT-guided or freehand technique. The surgery sequence was also randomly assigned by a random number chart. In the PDT group, PDT A was placed by hand and compressed slightly to the anterior surface of C2. K-wires with diameters of 2.0 mm and 2.5 mm were inserted approximately 5 mm in depth through the middle two guide tubes of PDT A. Another two 2.0-mm-diameter K-wires were guided through the lateral two guide tubes to drill the entry points of C2TOPI at approximately 1 mm in depth (Fig. 4a). A TRS was then screwed into the lower 2.5-mm-diameter hole. After the anterior arch of C1 and anterior prominent edges of the bilateral C2 facets were resected, the upper two holes of the TARP were fixed to C1 with lateral mass screws (Kangli Orthopedic Instrument, Jiangsu, China) (Fig. 4b). A special reduction instrument was then used to relocate C1 and C2 by applying the force between the TARP and TRS (Fig. 4c). When the inferior border of the TARP was adjusted to the height as high as the midpoint of the TRS, and the two entry points of C2TOPI were detected at the center of the pedicle screw holes of the TARP, the inferior two holes of the TARP were fixed with a VBS (Fig. 4d). After removing the TRS, PDT B was placed to fit the anterior surface of C2 that was not covered by the TARP, while its two short pins were inserted into the bony hole that was previously prepared. A 2.5-mm-diameter K-wire was then inserted into the C2 pedicle with the assistance of PDT B (Fig. 4e). C2TOPI was completed after the two 3.5-mm-diameter screws (Kangli Orthopedic Instrument, Jiangsu, China) were inserted (Fig. 4f). In the freehand group, C2TOPI was performed by the same attending surgeon using the fluoroscopy-guided freehand technique described in previous publications [1, 21]. The two 3.5-mm-diameter screws (Kangli Orthopedic Instrument, Jiangsu, China) were then inserted into the C2 pedicle. The surgery time for each group was recorded.

### Comparison of screw placement accuracy between the two groups

The cadaveric cervical specimens were scanned postoperatively with the same CT scanner using the same parameters. 3D reconstructions of the cadaveric C2 vertebrae with the inserted screws were also created with the same procedures. The screw insertion accuracy assessment was performed using Geomagic Studio 11.0 software (3D Systems Corp, Morrisville, NC). The postoperative centroids of the inserted screw at the midpoint of the pedicle in both the PDT and freehand groups were extracted.

The deviations of the centroids on the axial and sagittal planes of the pedicle between the preoperative design and postoperative screw position were calculated. The axial plane deviations towards the lateral side were recorded as positive values, and the deviations towards the medial side were recorded as negative values. The sagittal plane deviations towards the superior and inferior sides were recorded as positive and negative values, respectively.

Then, the pedicle screw positions were graded according to the modified All India Institute of Medical Sciences outcome-based classification [1, 2, 22]:

- Type I: ideal placement—screw threads are completely within the bony cortex
- Type II: acceptable placement—less than 50% of the diameter of the screw violates the surrounding cortex
- Type III: unacceptable placement—clear violation of the transverse foramen or spinal canal

### Statistical analysis

Statistical analysis was performed with SPSS 20.0 software (IBM Corporation, Armonk, NY, USA). Independent samples *t* tests were used to analyze the absolute values of the deviations between the two groups on the axial and sagittal planes as well as the surgery time. The chi-squared test was performed to compare the pedicle screw position between the two groups. A *p* value < 0.05 was considered statistically significant.

## Results

The PDTs were constructed successfully using 3D reconstruction and 3D printing. During the operation, the PDTs were fitted to their corresponding anterior cervical surfaces appropriately without any free movement. K-wires were easily inserted into the cervical pedicle with the assistance of the PDTs. The surgery time was 37.8 ± 4.5 min in the PDT group and 63.3 ± 3.82 min in the freehand group, resulting in a significant difference between the two groups (*t* = − 13.5, *p* < 0.001). The production time for this grouped PDT was approximately 70 min, and the manufacturing cost was approximately $30 per patient.

The absolute deviations from the centroids between the preoperative designs and postoperative measurements on the axial plane of the pedicle were 0.79 ± 0.64 mm in the PDT group and 1.69 ± 0.54 mm in the freehand group. On the sagittal plane of the pedicle, the corresponding values were 0.96 ± 0.51 mm in the PDT group and 1.72 ± 0.64 mm in the freehand group. Significant differences in the absolute deviations were observed on both the axial and sagittal planes (*t* = − 3.36, *p* = 0.003 and *t* = − 2.88, *p* = 0.01, respectively) (Table 1).

**Table 1:** Absolute deviations of screw trajectories at pedicle centroids

	PDT group	Freehand group
Axial plane (mm)	0.79 ± 0.64	1.69 ± 0.54
Sagittal plane (mm)	0.96 ± 0.51	1.72 ± 0.64

**Fig. 5:**
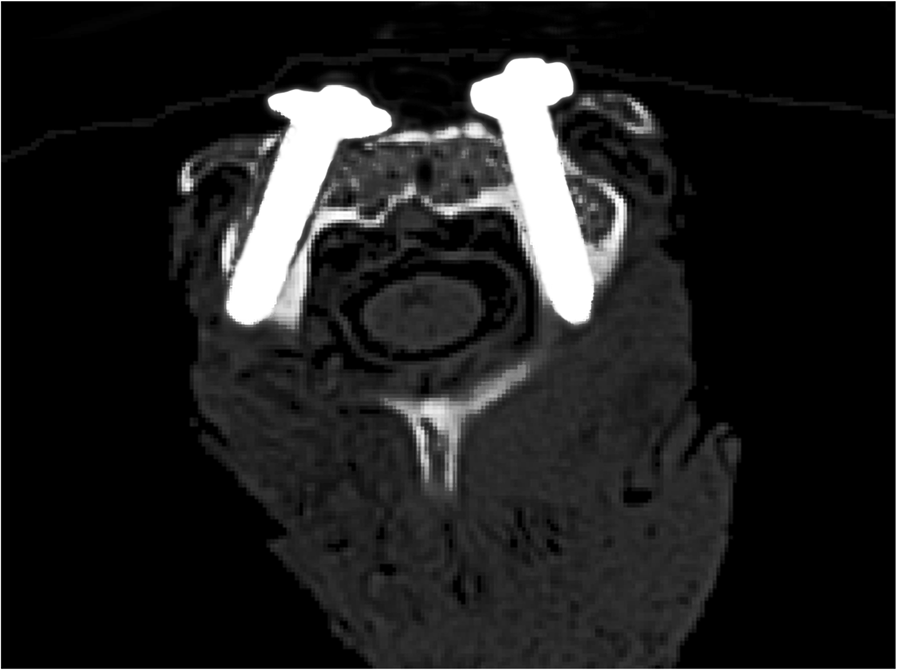
Postoperative CT scan showing good positioning of the pedicle screws (axial view)

Eight (80%) screw positions were type I, and two (20%) were type II in the PDT group. In the freehand group, five (50%) pedicle screw positions were type I, three (30%) were type II, and two (20%) were type III (Fig. 5). The classification of the screw positions was not significantly different between the two groups (*χ*^2^ = 2.553, *p* = 0.443) (Table 2).

**Table 2:** Classification of screw insertion

Grade	PDT group	Freehand group
Type I	8 (80%)	5 (50%)
Type II	2 (20%)	3 (30%)
Type III	0	2 (20%)

## Discussion

In this study, we developed a grouped PDT specifically for C2TOPI in AAD with complete reduction; the efficacy and veracity were further validated by comparison to the fluoroscopy-guided freehand technique.

In this study, we mainly focused on C2TOPI for AAD with complete anatomical reduction. An ideal trajectory that is considered to not only takes into account screw insertion safety but also biomechanical properties could be preoperatively designed for this procedure. A PDT might be subsequently developed to facilitate C2TOPI. However, unlike the usual lock-and-key structures of PDTs using a massive bone surface [23–25], this PDT was quite different. The TARP was not only an implant but also a reduction tool as well. After reduction of AAD, the TARP was temporally fixed with a VBS and covered most areas of the C2 anterior surface. Therefore, the PDT had two functions in facilitating C2TOPI. One was to facilitate an intraoperative reduction that allowed the TARP to find the ideal trajectory that was preoperatively designed. PDT A was designed for this purpose and allowed determination of the positions of the TRS, setscrew, and two C2TOPI entry points. In the intraoperative reduction, when the position of the bottom of the TARP reached the height of the TRS and the two entry points of C2TOPI were detected at the center of the pedicle screw holes of the TARP, the TARP was considered to have found the ideal trajectory and was then fixed with a VBS. The other purpose was to facilitate trajectory drilling that allowed the TARP to be fixed to C2 using transoral pedicle fixation. Because the TARP covered most of the area of the anterior surface of C2, the narrow uncovered surface, including the surface between two wings of the TARP and under the TARP, was used for location registration. PDT B, which included a table, the beneath registration columns/pins, and the above two guide tubes, was specially designed. PDT B was firmly attached to the anterior surface of C2 with good surface registration between the lower structures of columns/pins and the narrow surface not covered by the TARP. Trajectory drilling was then successfully performed using the guide tube. In the present study, the surgery time was approximately 1 h in the freehand group, which is slightly longer than most routine operations performed in the operation room. This result might have occurred because the surgeon was only an attending spinal surgeon with minimal experience in C2TOPI. He had to perform numerous fluoroscopic examinations to determine the ideal trajectory for C2TOPI; this required a lengthy time. However, for the same surgeon, the surgery time was much shorter in the PDT group. This finding indicates that the PDT could effectively improve the efficiency of young surgeons during their early attempts of C2TOPI. Further advantages of the PDT included reduced radiological exposure and the elimination of the need for complex equipment and complicated intraoperative procedures [16], especially for those with less C2TOPI experience.

The accuracy of PDTs in facilitating C2TOPI is of vital importance. In the present study, we calculated the absolute value of the deviations from the centroids between the preoperative designs and postoperative measurements, which had been used in our previous studies [25–27] to show the actual deviations. Our study showed that the absolute deviations in the axial plane (0.79 ± 0.64 mm) and the sagittal plane (0.96 ± 0.51 mm) in the PDT group might be within an acceptable range for clinical application. Both of these results were significantly different from those in the freehand group.

The rank index of the screw position in C2TOPI is also very important and can be used to assess critical cortex penetration intuitively. A rank index of the screw insertion position was introduced to evaluate the outcome of C2TOPI [1, 2]: type II is considered an acceptable breach, and a type III perforation is considered an unacceptable breach. This classification might be reasonable. Anatomical research has shown that the tolerance distances for C2TOPI consist of a space of approximately 1.1 mm between the lateral pedicle wall and vertebral artery and a space more than 1.1 mm between the medial pedicle wall and dural sac [28, 29]. Further clinical research has confirmed that perforations less than 50% of the diameter of the screw pose no risk of neurovascular injury. In our study, although no significant difference was observed in the screw positions between the two groups, two unacceptable breaches (type III) occurred in the freehand group (20%). This rate was slightly higher than that in previous reports (13.0%) [1], most likely because the surgeon had limited experience in AAD treatment, and the sample size was relatively small. In general, the results including the intuitionistic rank and quantitative absolute values of the deviations revealed that the PDT-guided technique was more precise than the fluoroscopy-guided freehand technique in facilitating C2TOPI.

Although the specially designed PDT had obvious advantages in facilitating C2TOPI, some limitations still exist and may require further attention. First, we used the ideal trajectory in our study. This trajectory was only considered for morphological safety but was not considered to be biomechanically optimized. Thus, further research is needed. Second, the PDT involved in this study was designed only for facilitating complete reduction of AAD in C2TOPI. A different strategy is required for incomplete reduction of AAD because the trajectory cannot be determined preoperatively. We designed a different PDT for this procedure that will be reported at a later time. Finally, the PDT was only used on cadaveric spines. Clinical studies are needed.

## Conclusion

In summary, specific PDTs could provide surgeons with an accurate and easy-to-apply method to facilitate C2TOPI for AAD with complete reduction. This could provide a viable alternative for surgeons.

## Abbreviations

3D: Three-dimensional
AAD: Atlantoaxial dislocation
C2TOPI: Transoral C2 pedicle insertion
PDT: Patient-specific drill template
TARP: Transoral atlantoaxial reduction plate
TRS: Temporary reduction screw
VBSs: Vertebral body screws
